# Downregulated liver-elevated long intergenic noncoding RNA (LINC02428) is a tumor suppressor that blocks KDM5B/IGF2BP1 positive feedback loop in hepatocellular carcinoma

**DOI:** 10.1038/s41419-023-05831-y

**Published:** 2023-05-03

**Authors:** Xuanlong Du, Pengcheng Zhou, Haidong Zhang, Hao Peng, Xinyu Mao, Shiwei Liu, Wenjing Xu, Kun Feng, Yewei Zhang

**Affiliations:** 1 School of Medicine, Southeast University, Nanjing, 210009 China; 2 Hepatopancreatobiliary Center, The Second Affiliated Hospital of Nanjing Medical University, Nanjing, 210011 China

**Keywords:** Liver cancer

## Abstract

Hepatocellular carcinoma (HCC) is a common malignant tumor with high mortality and poor prognoses worldwide. Many studies have reported that long noncoding RNAs (lncRNAs) are related to the progression and prognosis of HCC. However, the functions of downregulated liver-elevated (LE) lncRNAs in HCC remain elusive. Here we report the roles and mechanisms of downregulated LE LINC02428 in HCC. Downregulated LE lncRNAs played significant roles in HCC genesis and development. LINC02428 was upregulated in liver tissues compared with other normal tissues and showed low expression in HCC. The low expression of LINC02428 was attributed to poor HCC prognosis. Overexpressed LINC02428 suppressed the proliferation and metastasis of HCC in vitro and in vivo. LINC02428 was predominantly located in the cytoplasm and bound to insulin-like growth factor-2 mRNA-binding protein 1 (IGF2BP1) to prevent it from binding to lysine demethylase 5B (KDM5B) mRNA, which decreased the stability of KDM5B mRNA. KDM5B was found to preferentially bind to the promoter region of IGF2BP1 to upregulate its transcription. Therefore, LINC02428 interrupts the KDM5B/IGF2BP1 positive feedback loops to inhibit HCC progression. The KDM5B/IGF2BP1 positive feedback loop is involved in tumorigenesis and progression of HCC.

## Introduction

HCC accounts for 90% of primary liver cancers and is a global health challenge as the fourth leading cause of cancer-related death [1]. Major HCC risk factors include cirrhosis, hepatitis B, hepatitis C, and nonalcoholic steatohepatitis [2]. Patients with HCC (80%) are predominantly in sub-Saharan Africa and eastern Asia and their primary risk factors are chronic hepatitis B and aflatoxin B1 exposure [3]. The pathological mechanisms underlying HCC are complex, and the only way can we completely conquer this disease is by illustrating these mechanisms. Continuous efforts are being taken to achieve this goal worldwide. The symptoms often do not appear until a patient with HCC reaches an advanced stage; symptoms include abdominal pain, unexplained weight loss, and appetite descent [4]. Current clinical diagnosis methods of HCC have been improved, but some limitations remain. The early diagnosis of HCC is not ideal. Therefore, specific prognostic biomarkers are essential for early HCC diagnosis and treatment.

LncRNAs are a type of nonprotein coding transcript having a length of >200 nucleotides, which play an important role in HCC development and progression [5, 6]. With the development of next-generation sequencing, a large number of lncRNAs have been discovered and can function as oncogenes or tumor suppressors [7, 8]. LncRNAs have powerful functions, similar to proteins, and can participate in various molecular regulations in HCC. There is an interesting class of lncRNAs that is highly expressed in liver tissues compared with other normal tissues. Thus, they may maintain the characteristics of liver tissues. Their expression in HCC is low, so their HCC-related functions may have anticancer effects. We named this class of lncRNAs as downregulated liver-elevated (LE) lncRNAs.

Epigenetics refer to the change of heritable genetic expression without DNA sequence alteration through epigenetic modifications, including DNA methylation, histone modifications, noncoding RNAs, and three-dimensional chromatin organization [9]. RNA m6A modification is the most abundant methylation modification and plays an important role in RNA regulation [10]. The modification is reversible and catalyzed by corresponding enzymes, including m6A methyltransferases (“writers”), demethylases (“erasers”), and m6A-specific binding proteins (“readers”) [11]. m6A modifications regulate the fate of mRNA by influencing their interactions with m6A “reader” proteins [12]. The insulin-like growth factor-2 mRNA-binding protein 1 (IGF2BP1) is a member of the IGF2BP family (including IGF2BP1-3) and is an m6A “reader” protein [13]. IGF2BP1 is believed to promote the proliferation, migration, invasion, and metastasis of tumor cells [14]. Studies have shown that IGF2BP1 can regulate the stability of mRNA (including PTEN, ACTB, MAPK4, MKI67, c-MYC, and CD44 to affect tumor proliferation, migration, and invasion [12, 14–16]. Lysine demethylase 5B (KDM5B) is a histone lysine demethylase in histone modifications and facilitates the demethylation of tri-, di-, and monomethylated lysine 4 of histone H3 (H3K4) [17]. Similar to transcription factors, KDM5B regulates the transcription of the downstream genes. For example, Li G. et al. demonstrated that KDM5B directly binds the PIK3CA promoter to significantly positively regulate P110α and PIP3 [18]. Pu Y. et al. proved that KDM5B upregulated microRNA-448 to restrain papillary thyroid cancer cell progression [19].

We identified the prognostic value of the downregulated LE lncRNAs by retrospective analyses. We also explored the correlation of downregulated LE lncRNAs with tumor clinicopathological features. As a downregulated LE lncRNA, LINC02428 was selected to explore anticancer function in HCC.

LINC02428 was highly expressed in liver tissues but expressed at low levels in HCC and other normal tissues. LINC02428 was also associated with a good prognosis. LINC02428 acted as a tumor suppressor to inhibit HCC growth and metastasis. Furthermore, LINC02428 was found to interact with IGF2BP1 and interfere with KDM5B mRNA stability by blocking the combination between IGF2BP1 and KDM5B mRNA. We identified that KDM5B binds with the promoter region of IGF2BP1 to promote its transcription. Thus, a positive feedback loop between KDM5B and IGF2BP1 could drive HCC development. Therefore, these molecules could serve as promising therapeutic targets in HCC.

## Materials and methods

### Bioinformatic analyses

The RNA-seq data and corresponding clinical information (374 HCC and 50 normal samples) were downloaded from the Cancer Genome Atlas (TCGA) database (http://portal.gdc.cancer.gov/repository) on January 10, 2021. HCC and normal tissues were compared using the limma package from Bioconductor in R software (version 4.1.2), and differentially expressed (DE) lncRNAs were identified with the criteria for screening (FDR <0.05 and | log2FoldChange|>1).

DE downregulated LE lncRNAs were distinguished from low DE lncRNAs using the genotype-tissue expression (GTEx) database (https://www.gtexportal.org/home/). The correlation between expression levels of DE downregulated LE lncRNAs and patient overall survival (OS) was evaluated by univariate Cox regression analysis using the survival R package. LncRNAs with *P* < 0.05 univariate Cox regression analysis were considered as candidate variables.

Detailed methods of bioinformatics analyses are provided in Supplementary Methods.

### Patient and tissue samples

Tissue samples (carcinoma and adjacent tissue) were collected from 13 patients with HCC who had not received preoperative radiotherapy or chemotherapy at the Second Affiliated Hospital of Nanjing Medical University in 2022. Normal tissues were obtained from surgical specimens. The removed specimens were quickly placed in liquid nitrogen and stored at −80 °C for further analysis. The registered patients provided informed consent and the study was approved by the Second Affiliated Hospital of Nanjing Medical University Research Ethics Committee.

### Cell culture

HCC cell lines (Huh7, MHCC-97H) and a normal hepatic cell line (Lo2) were purchased from Xiamen Yimo Biological Technology Co. (Xiamen, China). Hep3B cells were purchased from Procell Life Science & Technology Co. (Wuhan, China), and HCC cell lines (SMHCC-7721, HCC-lm3, HepG2, and MHCC-97L) were provided by the First Affiliated Hospital of Nanjing Medical University. Huh7, MHCC-97H, SMHCC-7721, HCC-lm3, HepG2, and MHCC-97L were cultured in Dulbecco’s modified Eagle medium (Gibco, Grand Island, NY, USA) supplemented with 10% fetal bovine serum (FBS) (Gibco, Grand Island, NY, USA) and 1% penicillin-streptomycin (Beyotime, Shanghai, China), Hep3B was cultured in minimum essential medium (Gibco, Grand Island, NY, USA) supplemented with 10% FBS and 1% penicillin-streptomycin. Lo2 was cultured in Roswell Park Memorial Institute 1640 medium (Hyclone, Logan, UT, USA) supplemented 10% FBS and 1% penicillin-streptomycin. And all cell lines were incubated at 37 °C and 5% CO_2_. All cell lines were authenticated by STR profiling and confirmed to be mycoplasma negative.

### Small interfering RNAs (siRNAs), plasmids, lentivirus

siRNAs that specifically targeted KDM5B (Table S1) and overexpression plasmids (LINC02428, KDM5B, and IGF2BP1) were designed and synthesized by Sangon Biotech (Shanghai, China). siRNAs or plasmids were applied to transiently transfect HCC cells with Lipofectamine 2000 (Invitrogen, USA) following the manufacturer’s instructions. Lentivirus encoding LINC02428 and the negative control were purchased by GeneChem (Shanghai, China), These vectors were respectively transfected into HCC cells following the manufacturer’s instructions and stable cell lines were selected with puromycin for further study.

### RNA isolation and quantitative reverse transcription polymerase chain reaction (qRT-PCR)

The total RNA content of cells and tissues was extracted by TRNzol Universal (TIANGEN, Beijing, China), and extracted RNA was reversed into cDNA by the HiScript III RT SuperMix for qPCR (Vazyme, Nanjing, China). Specific and internal control primers were synthesized by Sangon Biotech (Shanghai, China), All primer sequences used in this study are listed in Table S1. qRT-PCR was performed to explore mRNA levels using ChamQ Universal SYBR qPCR Master Mix (Vazyme, Nanjing, China) and a real-time PCR system. The expression of glyceraldehyde‐3‐phosphate dehydrogenase (GAPDH) was used as a control for normalization and specific gene expression was calculated based on the 2^−ΔΔCt^ method.

### Cell proliferation assay

The cell proliferation assay of LINC02428 was performed using the Cell Counting Kit-8 (CCK-8) and 5-ethynyl-2′-deoxyuridine (EdU) assays. For the CCK-8 assay, cells were planted in 96-well plates at a density of 1000 cells per well. The CCK-8 reagent was purchased from Beyotime (Shanghai, China) and was added for 1, 2, 3, 4, and 5 days. After incubation for 3 h, the absorbance at 450 nm was detected using a microplate reader (BioTek ELx800 Absorbance Microplate Readers). For the EdU assay, BeyoClick™ EDU-488 Cell Proliferation Detection Kit was purchased from Beyotime (Shanghai, China). Cells with a density of ~1 × 10^4^ cells per well were seeded in a 96-well plate. The cultured cells were subjected to EdU labeling, fixation, washing, and nuclear staining as per the manufacturer’s instructions. Finally, the green fluorescence intensity and cell number were observed under a fluorescent microscope.

### Invasion and migration assays

For the invasion assay, the bottom membrane of the Transwell plates (24 wells and 8-μm pore size; Falcon) was coasted with diluted Matrigel matrix (Corning); however, this was not done for the migration assay. Serum-free medium containing 4 × 10^4^ cells (200 µL) were seeded in Transwell plates and 10% FBS medium (400 µL) was placed in the lower chamber. After 48 h of incubation at 37 °C, the Transwell plates were fixed with formaldehyde and stained with crystal violet for 15 min. The cells remaining on the upper surface of Transwell plates were removed with swabs, and cells of the lower surface were counted under a microscope.

### In vivo xenograft experiments

Animal experiments were carried out according to protocols approved by the Institutional Animal Care and Use Committee of Southeast University Medical School. For subcutaneous xenograft tumor models, Huh7 cells (4 × 10^6^) that were stably transfected with overexpression LINC02428 or scramble were subcutaneously implanted into 5-week-old male BALB/c nude mice that were randomly assigned to each group (*n* = 5 per group). The volume of tumor growth was monitored weekly (volume = length × width^2^ × 1/2). After 4 weeks, all mice were euthanized, and the tumors were collected for immunohistochemistry (IHC) analysis. For lung metastasis models, 1 × 10^6^ Huh7 cells (Lv-Vector or Lv-OELINC02428) were injected into the tail veins of nude mice (*n* = 5 per group). Six weeks after injection, the mice were sacrificed. Lung tissues were fixed in 4% paraformaldehyde for hematoxylin-eosin (HE) staining assay.

### Western blotting

The total protein content of cells was extracted with NP-40 lysis buffer (Beyotime) according to the manufacturer’s instructions. The total protein concentration was measured using a BCA Protein Assay Kit (Beyotime). An equivalent amount of total protein was separated by 10% sodium dodecyl sulfate-polyacrylamide gel electrophoresis (SDS-PAGE) and transferred onto a polyvinylidene fluoride (PVDF) membrane (Millipore, Burlington, USA). The PVDF membranes were blocked in 5% nonfat milk for 1 h at room temperature and incubated with primary antibodies at 4 °C overnight. Subsequently, the membranes were incubated with horseradish peroxidase-conjugated anti-mouse or anti-rabbit IgG at room temperature for 1 h. The signals were detected and visualized by BeyoECL Star (Beyotime). The following antibodies were used: anti-IGF2BP1 (22803-1-AP, Protientech; 1:3000 dilution), anti-KDM5B (15327 S, CST; 1:1000 dilution) and anti-GAPDH (GB15004, Servicebio; 1:2000 dilution).

### Fluorescence in situ hybridization (FISH) and immunofluorescence (IF) assays

The cells were subjected to the FISH using the FISH Kit (GenePharma, Shanghai, China) according to its manufacturer’s instructions. The design and synthesis of specific probes (Cy3-labeled 18S, NC and LINC02428 probes) were completed by GenePharma Company and the specific probes sequences can be found in Table S2. Following the FISH assay, cells were incubated with antibodies specific for IGF2BP1 (22803-1-AP, Protientech; 1:50 dilution) at 4 °C overnight. The cells were treated with Alexa Fluor® 488-conjugated Goat Anti-Rabbit IgG (GB25303, Servicebio; 1:250 dilution) at room temperature for 1 h and subjected to DAPI staining for 10 min. The cells were observed under a laser-scanning confocal microscope.

### RNA pull-down and RNA immunoprecipitation (RIP) assays

For the RAN pull-down assay, the Pierce™ Magnetic RNA-protein pull-down kit (2164, Thermo Fisher Scientific, USA) was used according to the manufacturer’s instructions. Briefly, biotin-labeled RNA was incubated with streptavidin magnetic beads under agitated conditions for 30 min at room temperature. Then, magnetic bead-labeled RNA molecules were incubated with cell lysates for 60 min at 4 °C. The RNA-binding protein complexes were separated by 10% SDS-PAGE and visualized by silver staining. For RIP assays, the Magna RIP™ RNA-Binding Protein Immunoprecipitation Kit (17–700, Millipore, USA) was applied according to the manufacturer’s instructions. Protein A/G beads were incubated with 5 μg of anti-IGF2BP1 (22803-1-AP, Protientech) and anti-IgG antibodies for 30 min at room temperature. The cell lysates were mixed with antibody-binding bead complexes at 4 °C overnight. Subsequently, proteinase K digested proteins and RNAs were extracted, purified, and subjected to reverse transcription and qRT-PCR.

### Chromatin immunoprecipitation (ChIP) and dual‑luciferase reporter assays

The ChIP assay was conducted using the SimpleChIP® Enzymatic Chromatin IP Kit (9003 S, CST, USA) according to the manufacturer’s instructions. In brief, DNA and proteins were crosslinked by 1% formaldehyde and chromatin was digested via micrococcal nuclease. Then, digested chromatin was incubated with 10 μg of anti-KDM5B (15327 S, CST) or anti-IgG antibodies at 4 °C overnight. The resulting antibody-chromatin complexes were incubated with 30 µl of protein G magnetic beads for 4 h. The chromatin was eluted, extracted, and analyzed using qPCR. The ChIP-related primers were shown in Table S1. The promoter of IGF2BP1 (−1000 to +361 bp) was subcloned and inserted into the pGL3-basic vector (Promega, Madison, USA) for dual‑luciferase reporter assay. Firefly luciferase reporter plasmid of IGF2BP1(pGL3-Basic-IGF2BP1-Pro-5′UTR) and ranilla luciferase reporter plasmid (pRL-TK) were co-transfected with overexpression plasmid of KDM5B or control vector to examine luciferase activities by Dual-Luciferase Reporter Gene Assay Kit (Genepharma, China). On the other hand, firefly, ranilla luciferase reporter, and the overexpression plasmid of KDM5B were co-transfected with the overexpressed plasmid of IGF2BP1 or LINC02428 to measure luciferase activities.

### IHC analyses

Tissue slides that were formalin-fixed and paraffin-embedded were dewaxed and rehydrated with graded ethanol and were blocked for endogenous peroxidase activity. Antigen slides were retrieved using a pressure cooker. The slides were incubated with 10% serum for 30 min at room temperature to block nonspecific antibody binding. Subsequently, the slides were incubated with anti-Ki-67 (AF1738, Beyotime; 1:100 dilution), anti-PCNA (AF0261, Beyotime; 1:200 dilution), anti-E-Cadherin (AF0138, Beyotime; 1:200 dilution), anti-N-Cadherin (22018-1-AP, Protientech; 1:2000 dilution) and anti- Vimentin (GB12192, Servicebio; 1:1000 dilution) at 4 °C overnight.

### RNA stability assay

Huh7 and Hep3B cells of overexpressed LINC02428 or vector cells were seeded into six-well plates and treated with 5 μg/ml actinomycin D (Sigma). The total RNA of cells was extracted at indicated time-points and mRNA expression of KDM5B was measured by qRT-PCR.

### Statistical analysis

The data downloaded from the TCGA database was processed by the Perl software (version 5.26.3). Statistical analysis and visualization were conducted using the R software (version 4.1.2) and GraphPad Prism 8. Differences between the two groups were analyzed using the independent Student’s t-test, whereas differences among multiple groups were analyzed using a one-way analysis of variance. The coefficients of downregulated LE lncRNAs were obtained using the LASSO algorithm. Pearson’s correlation analysis was used to explore correlations between the groups. Kaplan–Meier analysis and a log-rank test were performed for survival analysis. The accuracy and stability of the risk model were evaluated by receiver operating characteristic (ROC) curves and area under the curve (AUC) values. Paired student’s t-test was used the analyze LINC02428 expression in carcinoma and adjacent tissue. *P* value <0.05 was considered to be statistically significant. All experiments were performed in triplicates.

## Results

### Identification of downregulated LE lncRNAs in HCC

**Fig. 1:**
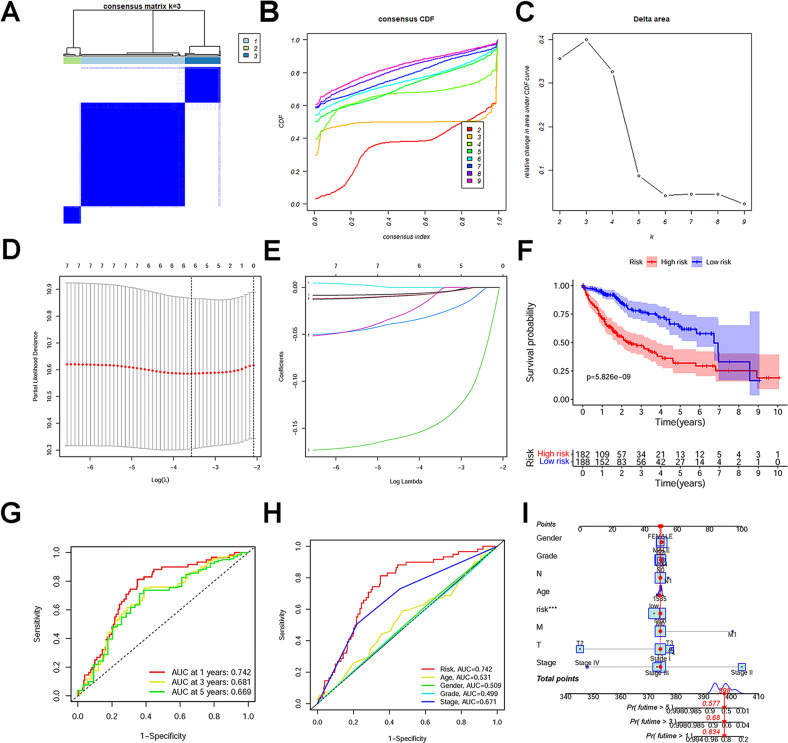
Bioinformatics analyses of LE lncRNAs. **A** A total of 370 patients with hepatocellular carcinoma (HCC) were divided into three clusters when K = 3. **B** Consensus clustering with cumulation distribution function (CDF) for K = 2–9. **C** Relative change in the area under CDF curve for K = 2–9. **D**, **E** Lasso Cox regression analysis of downregulated LE lncRNAs, cross-verification of the error curve based on the tuning parameters (log λ) of downregulated LE lcnRNAs, and a vertical dotted line at the optimal value by the minimal criterion. **F** Kaplan–Meier survival curves of the overall survival (OS) of patients with HCC in the high- and low-risk groups. **G** Receiver operating characteristic (ROC) curves and area under the ROC curve of 1-, 3-, and 5-year predictions. **H** C-index values of the risk score and clinicopathological characteristics. **I** The nomogram contains risk scores and clinical characteristics that predict 1-, 3-, and 5- years OS.

Using the limma package of R language for differential gene analysis (logFC ≥1 or ≤ −1, FDR <0.05), 6142 DE genes were obtained, of which 1575 were DE lncRNAs (including 1403 upregulated and 172 downregulated). Using the GTEx database, 49 LE lncRNAs were distinguished from 172 low DE lncRNAs in HCC (Table S3). Univariate Cox regression analysis was performed to analyze the correlations between the DE LE lncRNAs and the OS of HCC patients. Seven downregulated LE lncRNAs were significantly correlated with patient OS (*P* < 0.05) (Fig. S1A–G). The expression of seven downregulated LE lncRNAs in HCC and normal samples was shown by heatmap (Fig. S2A). Through consistent cluster analysis of the expression of seven downregulated LE lncRNAs in 370 patients with HCC, the 370 patients were finally divided into three subgroups (Fig. 1A–C). Though the Kaplan–Meier analysis, different clusters showed different OS (*P* < 0.05) the cluster1 of patients with HCC showed the worst prognosis compared to other clusters (Fig. S2D). As shown in the heatmap (Fig. S2C), the expression of downregulated LE lncRNAs and clinicopathological characteristics in different clusters was analyzed. Moreover, patients with HCC showing high expressions of lncRNAs were mostly concentrated in cluster2 and cluster3. This also confirmed why the prognoses of cluster2 and cluster3 were favorable. The expressions of AC008549.1 and AC115619.1 were the highest in cluster2 and cluster3, respectively. Cluster2 and cluster3 were significantly different from cluster1 in terms of T stage, grade, and stage, whereas the clusters showed no differences in sex and age (*P < 0.05 and **P < 0.01). By univariate Cox regression analysis, seven downregulated LE lncRNAs significantly correlated with OS were screened out from the TCGA dataset (Fig. S2B). Furthermore, six downregulated LE lncRNAs were used to establish a prognostic risk model by LASSO regression (Fig. 1D, E). The risk score = −0.00614 × LINC02428 + −0.00604 × AC008549.1 + −0.14553 × TMEM220-AS1 + −0.02841 × LINC02362 + −0.00601 × AC018467.1 + −0.00513 × AC115619. Patients with HCC were classified into high- and low-risk groups according to median risk scores. To verify the stability and accuracy of the risk model, Kaplan–Meier survival curve outcomes based on the median risk score show that the predicted survival time of the low-risk group was significantly longer than that of the high-risk group (*P* < 0.001; Fig. 1F). A time-dependent ROC curve was created to assess the predictive ability of the risk score to predict survival rates (Fig. 1G). The results showed that the 1-, 3-, and 5-year AUCs of the prognostic model were 0.742, 0.681, and 0.669, revealing that the risk model was performing stably. To test the predictive efficacy of the prognostic model, distribution diagrams of risk score, survival status, survival time, and expressions of six downregulated LE lncRNAs were visualized using the R software (Fig. S3F–H). These results provided further proof that patients with HCC and higher risk scores had lower OS than patients with lower risk scores. The high-risk group significantly differed from the low-risk group at the T stage, grade, and stage, whereas there were no differences in sex and age between the groups (Fig. S3B). PCA revealed that the six LE lcnRNAs participating in model construction could obviously separate patients from the high- and low-risk groups and compare the 49 downregulated LE lncRNAs and all genes (Fig. S3C–E). To further demonstrate the stability and accuracy of the risk model, the risk scores based the prognostic model can independently predict the prognosis of patients with HCC compared with other indicators by univariate and multivariate Cox regression analyses (Fig. 1H and Fig. S2E, F). Moreover, the nomogram containing risk scores and clinicopathological characteristics was constructed to predict the survival rates at 1, 3, and 5 years. Compared with clinical indicators, the risk scores showed significant predictive power (Fig. 1I). The calibration graphs showed a consistent prediction of 1-, 3-, and 5-year OS rates (Fig. S3A). We performed gene ontology (GO) and Kyoto Encyclopedia of Genes and Genomes (KEGG) functional enrichment analyses on co-expressed protein-coding mRNAs to investigate the potential functions of downregulated LE lncRNAs. The results indicated that co-expressed protein-coding mRNAs of selected six downregulated LE lncRNAs were enriched in 1003 GO terms, which primarily focused on immune cell regulation (Fig. S4A). KEGG functional enrichment analyses were significantly enriched in 67 KEGG pathways, primarily related to the metabolism of various substances and cell cycles (Fig. S4B). We have presented the top 30 GO/KEGG terms. This further indicated that downregulated LE lncRNAs may play a crucial role in HCC occurrence and development.

### Downregulated LE LncRNA LINC02428 showed low expression

**Fig. 2:**
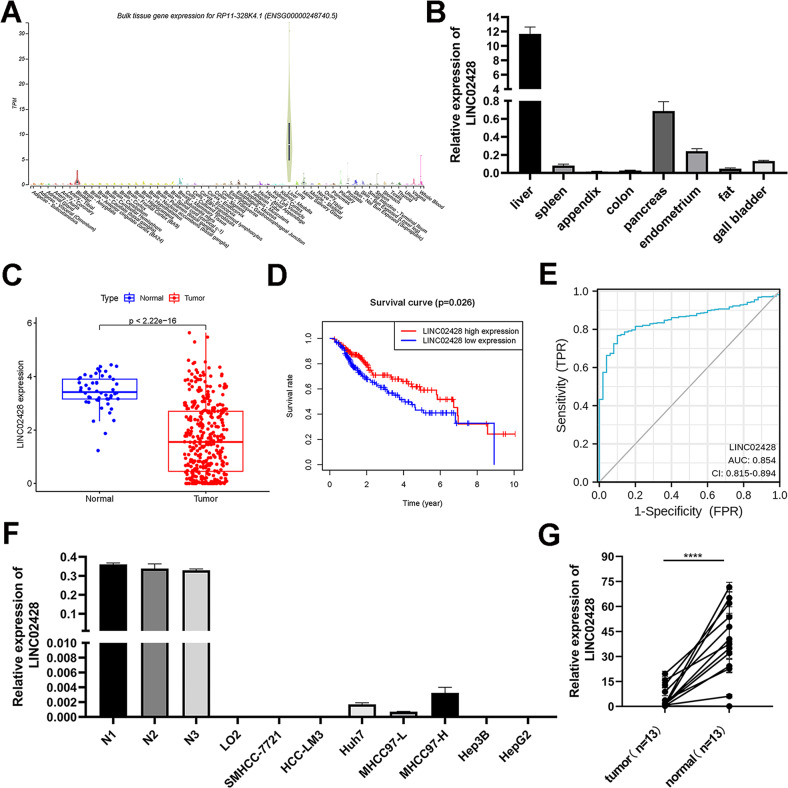
LINC02428 showed low expression with a good prognosis in hepatocellular carcinoma (HCC). **A** LINC02428 expression in 53 normal tissues from the GTEx database. **B** LINC02428 expression in eight normal human tissue, including the liver, spleen, appendix, colon, pancreas, endometrium, fat, and gall bladder. **C** LINC02428 expression in normal and patients with HCC from TCGA database. **D** Kaplan–Meier curve analysis of OS stratified according to LINC02428 expression in 370 patients with HCC from the TCGA database. **E** ROC curves of LINC02428. **F** LINC02428 expression in HCC cell lines. **G** RNA expression of LINC02428 in 13 pairs of HCC and adjacent tissues. (*P < 0.05, **P < 0.01, ***P < 0.001).

The roles of downregulated LE lncRNAs in HCC were explored. The role of LINC02428 in HCC remains elusive; thus, LINC02428 was selected from the six LE lncRNAs of the risk model. Among 53 normal tissues, LINC02428 was found to exhibit the highest expression in liver tissue from the GTEx database (Fig. 2A). qRT-PCR results of eight normal clinical tissue samples also showed that LINC02428 was highly expressed in the liver tissue (Fig. 2B). The high expression of LINC02428 in normal liver tissue was compared with the expression in HCC tissue samples (Fig. 2C) and survival analysis showed that patients with HCC exhibiting high expression of LINC02428 had improved prognoses through TCGA database (Fig. 2D). The AUC was 0.854 and the expression of LINC02428 could predict the prognoses of patients with high accuracy (Fig. 2E). The qRT-PCR results of HCC cell lines and 13 pairs of cancer and adjacent tissues from patients with HCC showed the expression of LINC02428 in HCC was low (Fig. 2F, G). High expression of LINC02428 in HCC may play an inhibitory role in HCC tumorigenesis and development.

### Downregulated LE lncRNA LINC02428 suppresses HCC proliferation and metastasis in vitro and in vivo

**Fig. 3:**
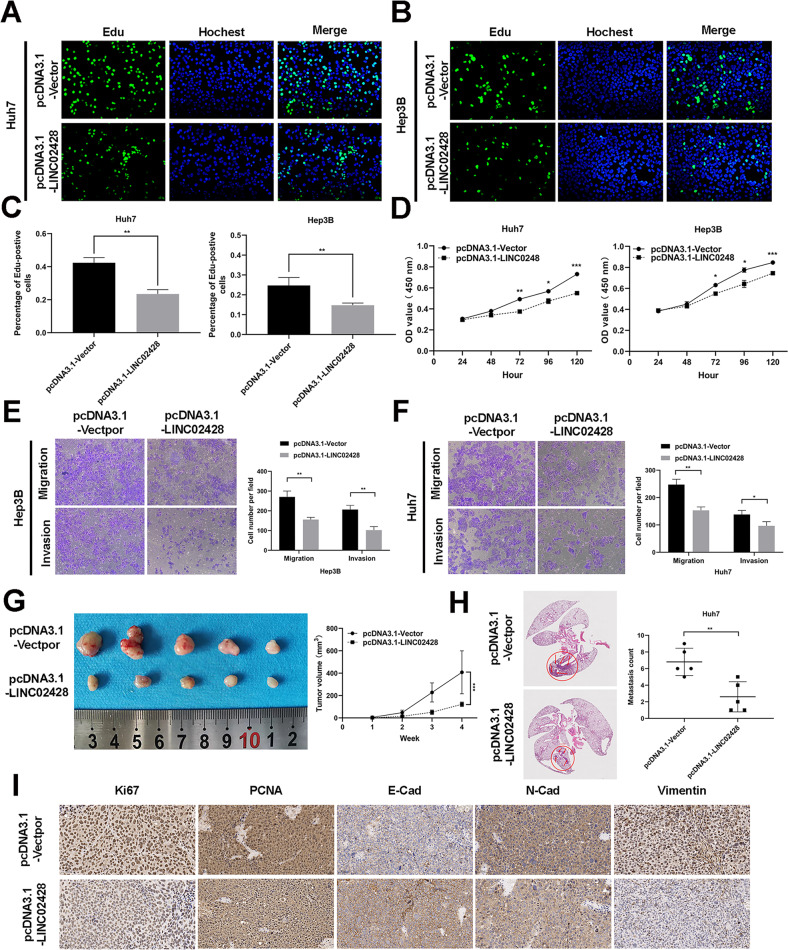
LINC02428 inhibits HCC cell proliferation and metastasis in vitro and vivo. **A**–**C** The EdU assay showed that LINC02428 overexpression reduced the rate of EdU-positive HCC cells. **D** The CCK-8 assay indicated that LINC02428 upregulation decreased HCC cell proliferation. **E**, **F** LINC02428 overexpression suppressed migration and invasion compared to a negative control. **G** Representative images of tumors from xenograft tumor models and the tumor growth curve. **H** Representative images of HE staining of lung metastases in nude mice with Huh7 cells and a scatter plot of the number of Lung metastasis. **I** immunohistochemistry images of Ki-67, PCNA, E-Cad, N-Cad, and Vimentin levels in subcutaneous xenografts.

To investigate the antitumor effect of LINC02428 in HCC, we established Huh7 and Hep3B cell lines by overexpression of LINC02428, and 50 times as many expressions in cells are LINC02428 expression as a control. (Fig. S5A). The EdU assay results showed that LINC024283 overexpression significantly suppressed HCC cell proliferation in vitro (Fig. 3A–C). The CCK-8 assay results also revealed significantly inhibited Huh7 and Hep3B cell proliferation by LINC02428 overexpression (Fig. 3D). The migration and invasion assay results indicated that Huh7 and Hep3B cells with LINC02428 overexpression exhibited low migration and invasion capacities compared with the negative control (Fig. 3E, F). Thus, these findings strongly showed that LINC02428 overexpression could repress HCC proliferation, migration, and invasion.

To explore the role of LINC02428 in vivo, Huh7 cells with stable LINC02428 overexpression or a normal control were subcutaneously injected into BALB/c nude mice. The tumor volume was considerably smaller in the LINC02428-overexpression group than that in the control group, implying that LINC0248 overexpression could inhibit tumor cell proliferation in HCC (Fig. 3G). The IHC staining assay results showed decreased levels of the proliferation markers Ki-67 and PCNA and the EMT-related markers N-cad and Vimentin and the increased level of E-cad in tumors with LINC0248 overexpression compared with those in the control group (Fig. 3I). Consistent with the IHC staining assay, the lung metastasis model also showed that LINC02428 overexpression could reduce HCC lung metastasis (Fig. 3H). Thus, LINC02428 overexpression could inhibit HCC growth and metastasis.

### Downregulated LE lncRNA LINC02428 binds to IGF2BP1 to decrease its mRNA and protein levels

**Fig. 4:**
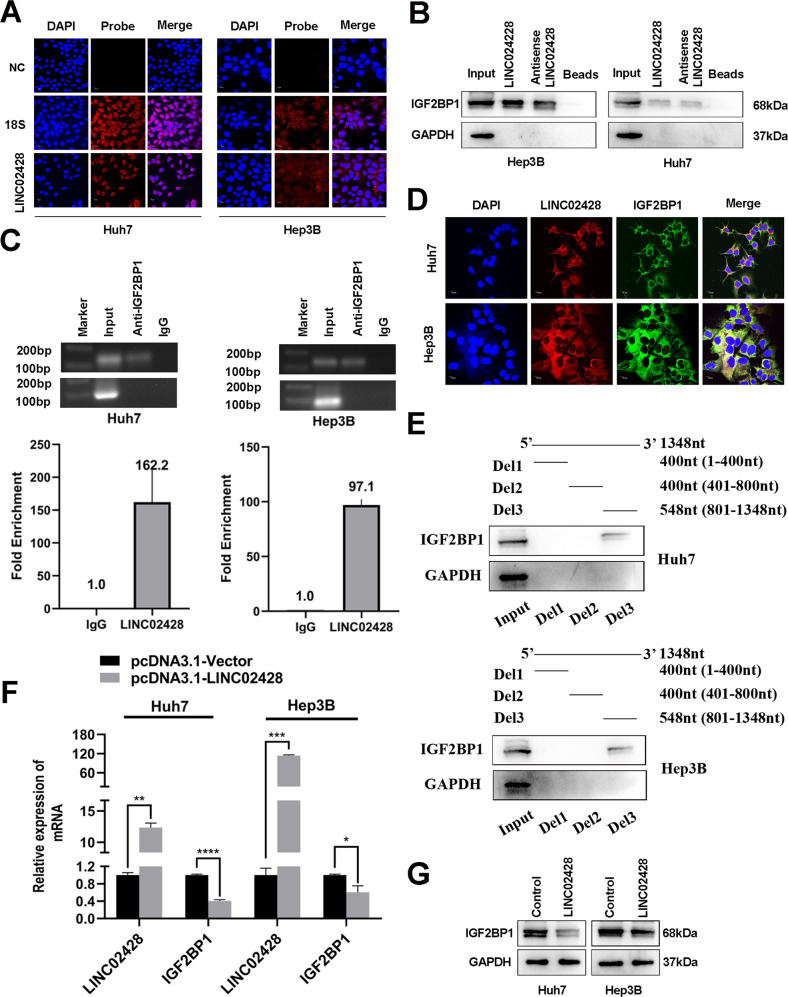
LINC02428 interacts with IGF2BP1 to affect the expression of IGF2BP1 mRNA and protein levels. **A** FISH assay showed that LINC02428 was mainly located in the cytoplasm of HCC cells**. B** Western blotting of proteins obtained by RNA pull-down assay showed that LINC02428 bound to IGF2BP1. **C** RIP assays confirmed that LINC02428 interacts with IGF2BP1. **D** FISH and IF assays show LINC02428 and IGF2BP1 are co-localized in the cytoplasm in HCC cells. **E** Deletion mapping of the IGF2BP1-binding domain in LINC02428 was examined by western blotting. **F** qRT-PCR showed LINC02428 upregulation decreased IGF2BP1 mRNA levels. **G** Western blotting indicated LINC02428 overexpression decreased IGF2BP1 protein levels.

To elucidate the underlying mechanisms of LINC02428 in HCC, we first performed FISH assays to find that LINC02428 was mainly located in the cytoplasm (Fig. 4A). The results showed that LINC02428 could interact with some proteins to exert its antitumor effect in the cytoplasm. RNA pull-down assay was performed; however, differential bands were not observed for the sense and antisense strands of LINC02428 in Huh7 cells (Fig. S5B). However, the antisense strand of lncRNAs is often not a good control for RNA pull-down assays; thus, the absence of differential bands does not imply the absence of specific binding proteins. We found that both the sense and antisense strands of LINC02428 could bind to IGF2BP1 using RBPsuite (http://www.csbio.sjtu.edu.cn/bioinf/RBPsuite/) (Fig. S5C, D) [20, 21]. Thus, we performed western blotting using an anti-IGF2BP1 antibody to verify that IGF2BP1 existed in eluted samples of LINC02428 sense and antisense stands obtained after the pull-down assay (Fig. 4B). On the other hand, RIP assays results indicated that LINC02428 was dominantly enriched in the IP-RNA of IGF2BP1 compared with IgG (Fig. 4C). Concurrently, FISH and IF assay results showed that LINC02428 and IGF2BP1 were co-localized in the cytoplasm of HCC cells (Fig. 4D). In summary, these results suggested that LINC02428 was specifically bound to IGF2BP1. To validate the binding regions between LINC02428 and IGF2BP1, we performed RNA pull-down assays of truncated LINC02428 fragments and cell lysates of Huh7 and Hep3B cells. Deletion mapping analysis indicated that the 801- to 1348-nt region of LINC02428 was indispensable for interacting with IGF2BP1 (Fig. 4E).

We further demonstrated whether LINC02428 regulated IGF2BP1 in HCC, we first found that LINC02428 expression was negatively correlated with IGF2BP1 mRNA expression by performing gene expression profiling interactive analysis (GEPIA) (http://gepia.cancer-pku.cn/index.html) (Fig. S5E) [22]. We probed mRNA and protein levels of IGF2BP1 in HCC cells overexpressing LINC02428 (Huh7 and Hep3B). The results indicated that LINC02428 overexpression could downregulate mRNA and protein levels of IGF2BP1 in HCC cells (Fig. 4F, G).

### KDM5B/IGF2BP1 positive feedback loop is antagonized by LINC02428

**Fig. 5:**
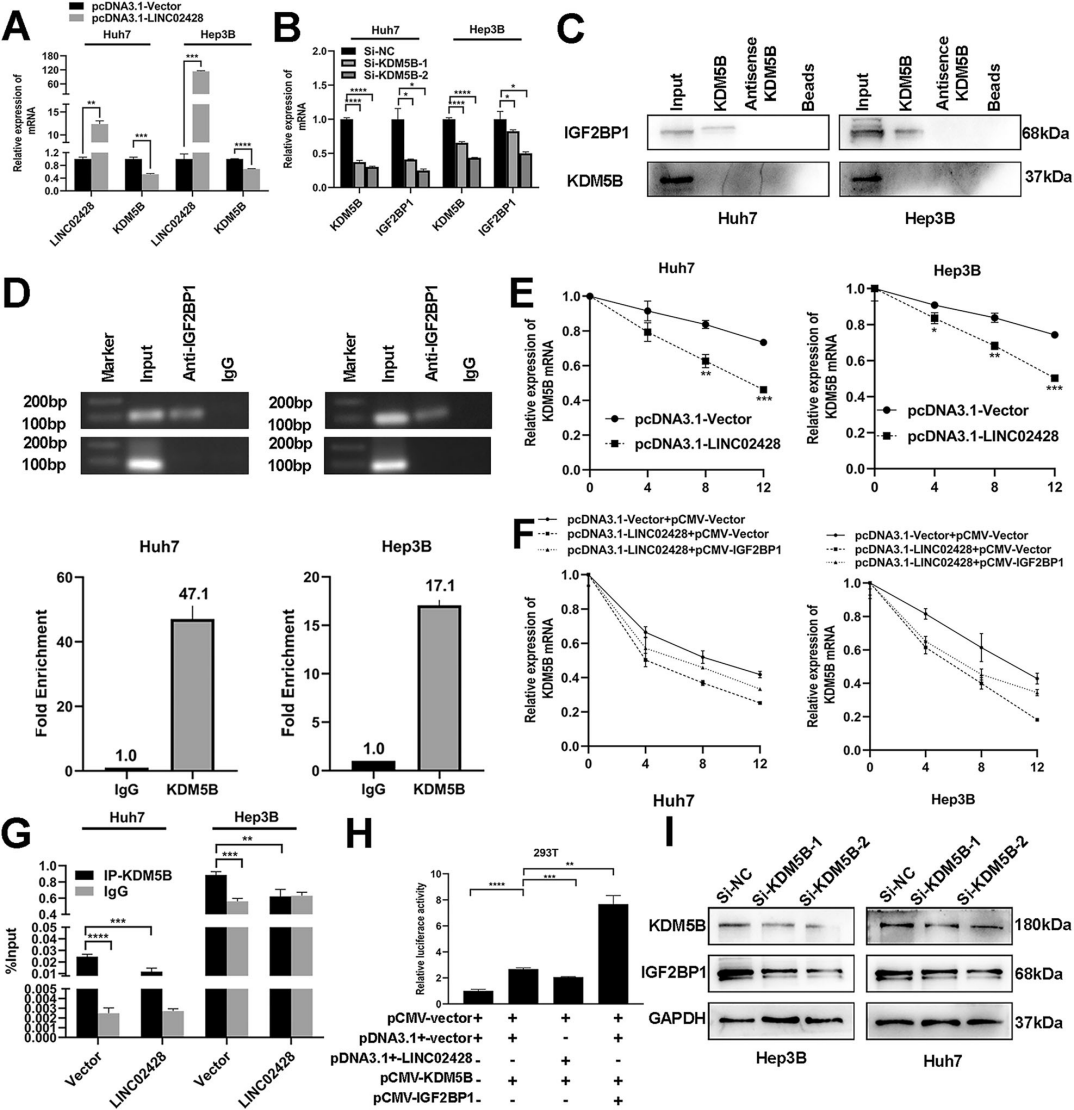
LINC02428 blocked the KDM5B/IGF2BP1 positive feedback loop in HCC cells. **A** Increased LINC02428 expression resulted in decreased KDM5B mRNA expression. **B** KDM5B downregulation decreased IGF2BP1 mRNA levels. **C** The protein lysate of KDM5B mRNA pull-down contained IGF2BP1. **D** RIP assays showed that IGF2BP1 could recruit KDM5B mRNA. **E** RNA stability assay indicated that LINC02428 overexpression could weaken the stability of KDM5B mRNA. **F** IGF2BP1 upregulation could reverse the LINC02428-mediated decrease in KDM5B mRNA stability. **G** ChIP-qPCR assay confirmed that KDM5B enriched in the promoter of IGF2BP1 and LINC02428 upregulation could decrease KDM5B enrichment. **H** Dual luciferase reporter assay proved that KDM5B bound to IGF2BP1 promoter to promote its transcription, and IGF2BP1 transcription promoted KDM5B could be enhanced by upregulation of IGF2BP1 or decrease by LINC02428 overexpression in 293 T. **I** Western blotting showed that KDM5B silencing could decrease expression of IGF2BP1 protein in HCC cells.

Many studies have shown IGF2BP1 as a post-transcriptional fine-tuner that can enhance mRNA stability and translation to regulate the development and progression of tumor cells. Here, we found that LINC02428 binding to IGF2BP1 decreased IGF2BP1 mRNA and protein levels. We speculated that LINC02428 binding to IGF2BP1 blocked the function of IGF2BP1 in regulating mRNAs stability. Hence, we searched for a molecule whose mRNA might interact with IGF2BP1 to upregulate stability and whose protein might play a role in the transcriptional regulation of IGF2BP1. The GEPIA result showed that KDM5B mRNA level was positively correlated with IGF2BP1 (Fig. S5G), whereas it was negatively correlated with LINC02428 in HCC (Fig. S5H). The qRT-PCR results were consistent with these results. LINC0248 overexpression downregulated the KDM5B mRNA level in Huh7 and Hep3B cells (Fig. 5A), and silencing KDM5B downregulated the IGF2BP1 mRNA level (Fig. 5B). The RIP-seq of IGF2BP1 from the NCBI GEO database showed that KDM5B mRNA interacted with IGF2BP1 (GSE196238). The RNA pull-down and RIP assays of KDM5B also showed that KDM5B mRNA could specifically bind to IGF2BP1 in Huh7 and Hep3B cells (Fig. 5C, D). The mRNA stability assay results indicated that LINC02428 overexpression decreased KDM5B mRNA stability in HCC cells (Fig. 5E). To further clarify whether LINC02428 blocked the recruitment of IGF2BP1 to KDM5B mRNA and affected KDM5B mRNA stability, we performed a rescue experiment where IGF2BP1 and LINC02428 were simultaneously overexpression in Huh7 and Hep3B cells. We found that the decreased KDM5B mRNA stability because of LINC02428 overexpression was partially restored by ectopic IGF2BP1 expression in the Huh7 and Hep3B cells (Fig. 5F). Altogether, these results showed that LINC02428 overexpression antagonized IGF2BP1 binding to KDM5B mRNA and downregulated the stability of KDM5B mRNA.

We obtained peak areas enriched in 0–300 nt upstream of the IGF2BP1 promoter region in most ChIP-Seq data on KDM5B (Fig. S6A) using Cistrome Data Browser (http://cistrome.org/db/#/) and ChIPBase v2.0 (https://rna.sysu.edu.cn/chipbase/index.php) showed that KDM5B interacted with the binding site at 75 nt upstream of the IGF2BP1 promoter (Fig. S6B). Therefore, ChIP primers were designed for ChIP-PCR at 0–300 nt upstream of the IGF2BP1 promoter. The results showed that KDM5B bound at 0–300 nt upstream of the IGF2BP1 promoter, and LINC02428 overexpression decreased KDM5B binding to the IGF2BP1 promoter region (Fig. 5G). The dual luciferase reporter assay results showed that KDM5B could regulate IGF2BP1 transcription, and luciferase activity decreased after LINC02428 overexpression, whereas it increased after IGF2BP1 overexpression in 293 T and Hep3B cells (Fig. 5H and S6C). Consistent with the dual luciferase reporter assay results, western blotting showed that silencing KDM5B reduced IGF2BP1 expression in Huh7 and Hep3B cells (Fig. 5I). These results suggested that KDM5B played a role in the activation of IGF2BP1 transcription in HCC.

Overall, the above results indicated the presence of a positive feedback loop between KDM5B and IGF2BP1 in HCC. KDM5B is a transcription promoter of IGF2BP1, and IGF2BP1 can stabilize KDM5B mRNA to increase KDM5B expression, thus forming a positive feedback loop in HCC. Moreover, this loop can be blocked by LINC02428 to inhibit HCC occurrence and development.

### Blocking the KDM5B/IGF2BP1 positive feedback loop mediates the effect of LINC02428 on tumor phenotypes

**Fig. 6:**
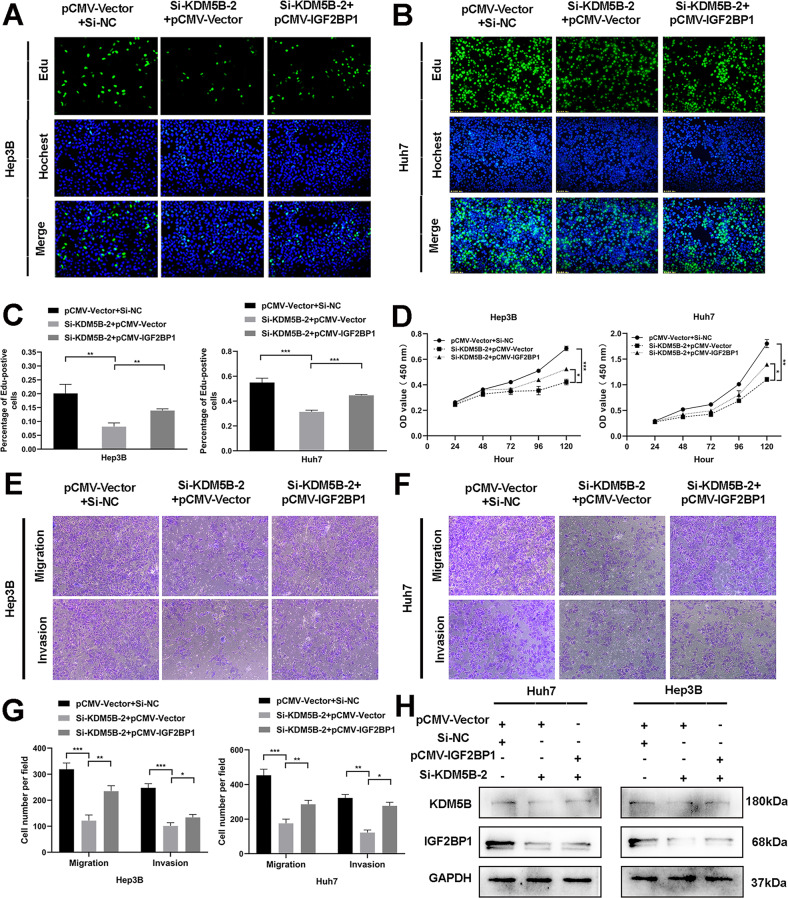
KDM5B affects tumor phenotypes via IGF2BP1. **A–C** EdU assay **D** CCK-8 assay indicated that KDM5B silencing could suppress HCC proliferation; however, IGF2BP1 upregulation could reverse the inhibition of proliferation. **E–G** IGF2BP1 overexpression rescued the KDM5B silencing-induced inhibition of migration and invasion in HCC cells. **H** Western blotting confirmed KDM5B knockdown downregulated IGF2BP1, whereas IGF2BP1 co-transfection reversed KDM5B downregulation.

**Fig. 7:**
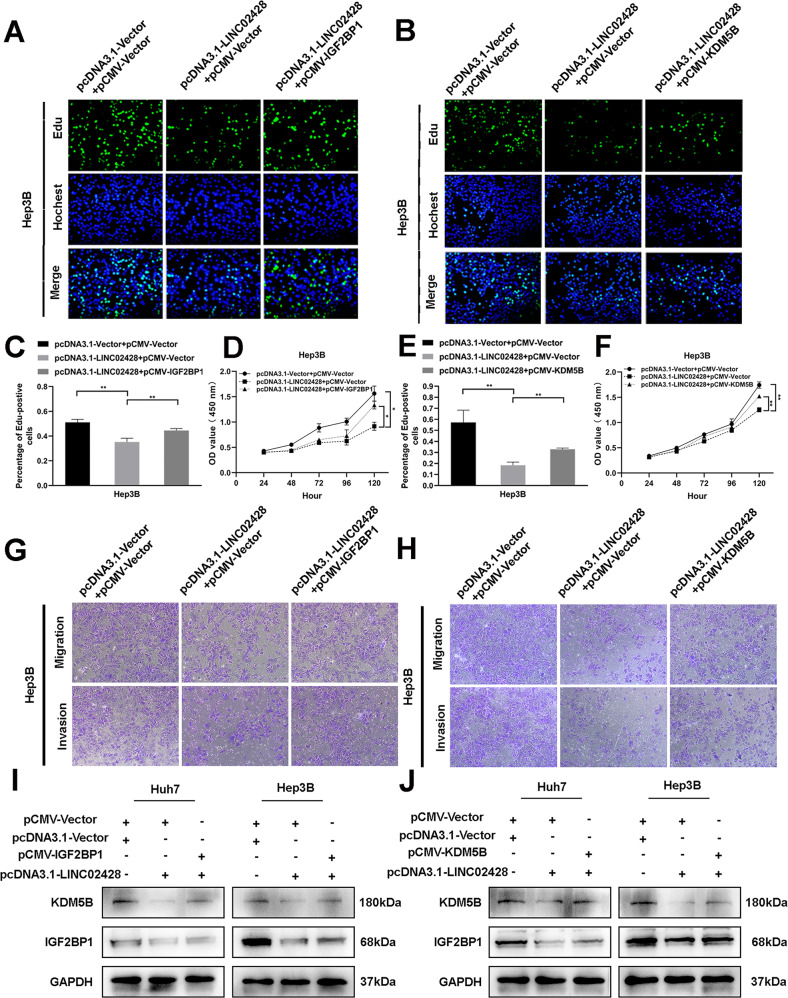
IGF2BP1 or KDM5B rescued antitumor phenotypes induced by LINC02428 overexpression in Hep3B cells. EdU and CCK-8 assays showed that the upregulation of IGF2BP1 (**A**, **C**, **D**) or KDM5B (**B**, **E**, **F**) impaired the overexpression of the LINC02428-induced suppression of HCC proliferation. Migration and invasion assays indicated that IGF2BP1 (**G**) or KDM5B (**H**) overexpression rescued the inhibition of HCC migration and invasion induced by LINC02428 upregulation. **I** Western blotting showed expression of protein about both upregulation LINC02428 and IGF2BP1. **J** Western blot assays showed protein levels after LINC02428 and KDM5B upregulation.

**Fig. 8:**
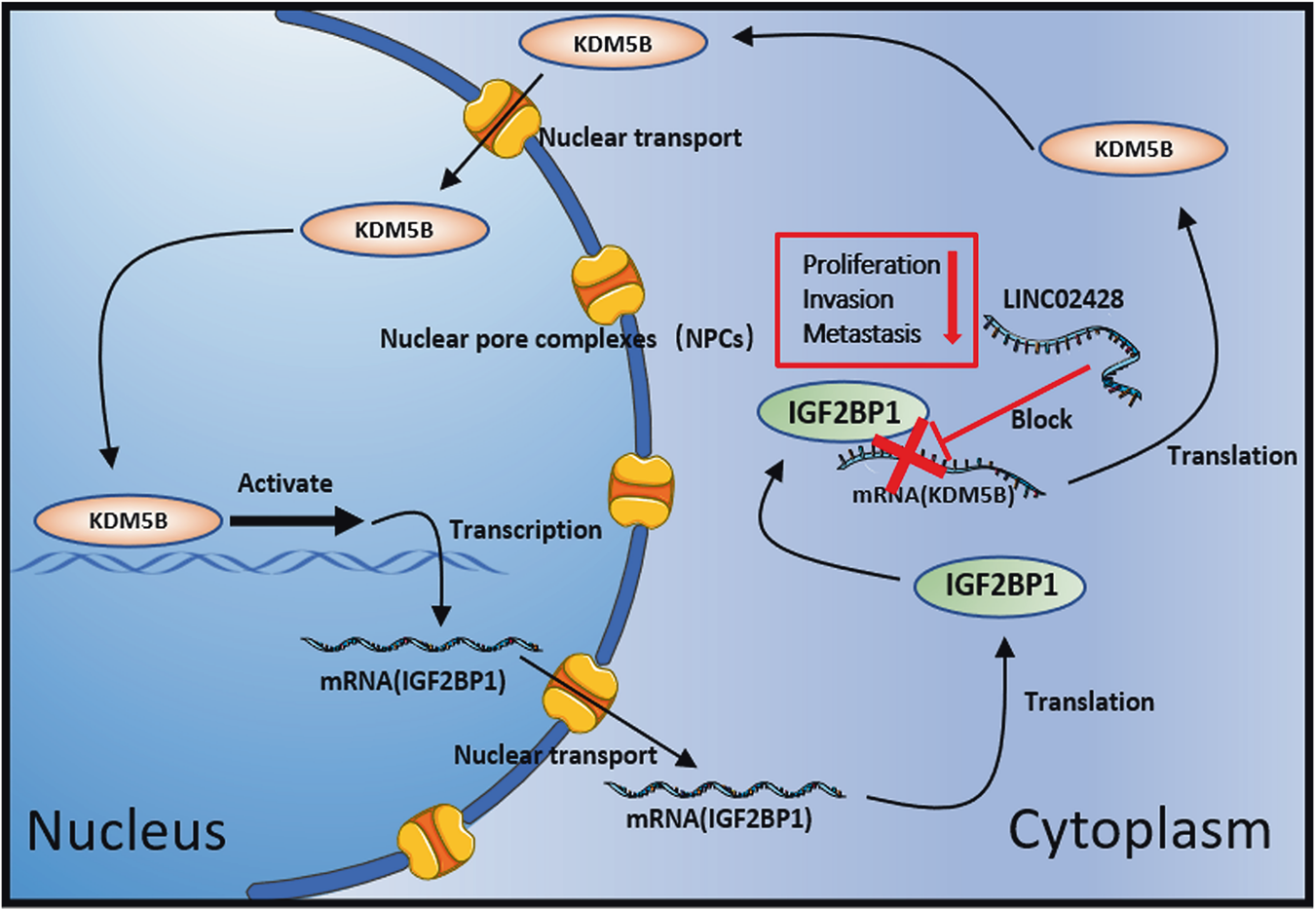
A schematic diagram. LINC02428 blocked the KDM5B/IGF2BP1 positive feedback loop to inhibit HCC progression.

We first performed rescue experiments to investigate the interplay between KDM5B and IGF2BP1 on HCC phenotypes. These results indicated that IGF2BP1 overexpression significantly impaired the silencing KDM5B-mediated decrease in cell proliferation (Fig. 6A–D), invasion, and migration in HCC cells (Fig. 6E–G). Furthermore, the western blotting results showed that IGF2BP1 overexpression could largely rescue the inhibition of KDM5B knockdown (Fig. 6H). Next, we transfected IGF2BP1-overexpression plasmids into LINC02428-overexpression Huh7 and Hep3B cells and showed that IGF2BP1 overexpression reversed the LINC02428-induced decrease in HCC cell proliferation (Fig. 7A, C, D and S7A, C, D), migration, and invasion in vitro (Fig. 7G and S7G, I, J). Western blotting indicated that IGF2BP1 resisted LINC02428 overexpression to upregulate KDM5B expression in HCC cells (Fig. 7I). Finally, we performed rescue experiments to explore whether LINC02428 exerted its effects via KDM5B. In vitro EdU and CCK-8 assays revealed that KDM5B upregulation partially decreased the LINC02428-induced decrease in HCC cell proliferation (Fig. 7B, E, F and S7B, E, F). Additionally, the LINC02428-mediated decreased invasion and migration capacities were partially rescued by KDM5B upregulation (Fig. 7H and S7H, K, L). Western blotting showed that KDM5B reversed LINC02428 overexpression to upregulate IGF2BP1 expression in Huh7 and Hep3B cells (Fig. 7J). These results showed that LINC02428 antagonized the KDM5B/IGF2BP1 positive feedback loop as a tumor suppressor in HCC (Fig. 8).

## Discussion

Emerging research has indicated that many lcnRNAs are associated with HCC prognosis [23]; thus, finding effective lcnRNAs for the diagnosis, treatment, and prognosis of HCC is essential. However, downregulated LE lncRNAs have been poorly studied with respect to HCC. In 2020, the LncRNA Spatial Atlas of Expression across Normal and Cancer Tissues (LncSpA) website was established to confirm the important role of tissue-elevated lncRNAs in cancer [24]. One year later, Xu, K. et al. performed a bioinformatics analysis and verified that the differential distribution of tissue-elevated lncRNAs in cancer and normal tissues was related to the regulation of m6A [25]. Cao, C. et al. demonstrated the inhibitory effect of TMEM220-AS1 on HCC via mir-484 /MAGI1 [26]. In the same year, Liu, Y. et al. also proved that TMEM220-AS1 inactivated the Wnt/β-catenin pathway and inhibited HCC [27]. LINC02499 and LINC01039, downregulated LE lncRNAs, were also proved to be excellent prognostic biomarkers and inhibitors of HCC [28, 29]. The previous study findings proved the inhibitory effect of downregulated LE lncRNAs in HCC; however, their mechanisms in HCC need further exploration.

In the present study, we first identified downregulated LE lncRNAs by performing consensus clustering analysis, survival analysis, constructing a prognostic risk model, and enrichment analysis and found that these LE lncRNAs played a vital role in HCC tumorigenesis, diagnosis, prognosis, and treatment. Then, we selected the downregulated LE lncRNAs LINC02428 with unknown function in HCC as a study subject. The present results indicated that LINC02428 expression was low in HCC cell lines and patients with HCC. LINC02428 exerted an antitumor effect in HCC, as indicated by CCK-8, EDU, invasion, and migration assays. In vivo experiments also showed that LINC02428 significantly inhibited HCC proliferation and metastasis.

LncRNAs mainly act as a “molecular sponge” to interact with miRNAs or to bind to a protein to affect protein functions [30]. In the present study, we demonstrated that LINC02428 could directly bind to IGF2BP1 and LINC02428 upregulation affected IGF2BP1 mRNA and protein levels. IGF2BP1, a key m6A reader, participates in the regulation of mRNA stability. Presently, many studies on IGF2BP1 in HCC are available. IGF2BP1 acts as a crucial upstream molecule by stabilizing downstream mRNAs to affect HCC occurrence and development [31]. For example, IGF2BP1/IGF2BP3 regulated the stability of lnc-CTHCC to promote hepatocellular carcinogenesis [32]. Rong, Z. et al. found that the LINC01134/miR-324-5p/IGF2BP1/YY1 feedback loop mediated HCC progression [33]. Here, we found that KDM5B was positively correlated with IGF2BP1, whereas it was negatively correlated with LINC02428. IGF2BP1 could bind to KDM5B mRNA and mediate the stability of KDM5B mRNA. LINC02428 upregulation could decrease the stability of KDM5B mRNA, and this change could be rescued by IGF2BP1 overexpression. KDM5B plays a vital role in transcriptional regulation by upregulating oncogenes or downregulating tumor suppressors [34]. In 2015, Shigekawa, Y. et al. first discovered that KDM5B was associated with a poor prognosis, and it positively regulated E2F1 and E2F2 transcription in HCC [35]. Wang D et al. proved that KDM5B overexpression regulated the histone H3K4 trimethylation of p15 and p27 promoters to inhibit their expression in HCC [36]. Chen B et al. also found that KDM5B attenuated FBXW7 transcription to affect the malignant proliferation of Ewing sarcoma [37]. In the present study, we determined that KDM5B directly bound to the promoter region of IGF2BP1 to promote IGF2BP1 expression, and a decrease in IGF2BP1 induced by LINC02428 overexpression could be reversed by KDM5B overexpression. In summary, we found that the novel KDM5B/IGF2BP1 positive feedback loop was antagonized by LINC02428 in HCC.

We revealed that downregulated LE lncRNAs exerted an antitumor effect on HCC. The downregulated LE lncRNAs LINC02428, a suppressor, inhibited HCC proliferation and metastasis by blocking the KDM5B/IGF2BP1 positive feedback loop. These results provide new insights into the mechanism of HCC progression and help develop therapeutic targets for HCC.

## Supplementary information

SUPPLEMENTAL MATERIAL

aj-checklist

## Data Availability

The datasets used and/or analysed during the current study are available from the corresponding author on reasonable request.
