# Supplementary material for: Downregulated liver-elevated long intergenic noncoding RNA (LINC02428) is a tumor suppressor that blocks KDM5B/IGF2BP1 positive feedback loop in hepatocellular carcinoma
Source: Cell Death Dis. 2023 May 3;14(5):301. doi: 10.1038/s41419-023-05831-y (PMC10156739; doi:10.1038/s41419-023-05831-y)
Supplement: Supplementary file 1 — SUPPLEMENTAL MATERIAL [file 41419_2023_5831_MOESM1_ESM.docx]

**Downregulated Liver-elevated Long Intergenic Noncoding RNA（LINC02428）is** **a Tumor Suppressor that Blocks** **KDM5B/IGF2BP1 Positive Feedback Loop in Hepatocellular Carcinoma**

Xuanlong Du, Pengcheng Zhou, Haidong Zhang, Hao Peng, Xinyu Mao, Shiwei Liu, Wenjing Xu, Kun Feng and Yewei Zhang

**Supplementary Information**

**Supplemental Methods:**

To further explore the potential functions of downregulated LE lncRNAs in HCC, HCC patients from TCGA database were clustered into different subgroups by the ConsensusClusterPlus R package with cycle computation 1,000 times to ensure stability and reliability. Principal component analysis suggested that the optimal k value (k=3) can obtain stable clustering. Differential analysis of subgroups and clinicopathological parameters was performed by chi-square test and survival analysis of different clusters was calculated using the Kaplan-Meier method.

We further selected downregulated LE lncRNAs through the LASSO regression algorithm to establish prognostic model. Eventually, coefficients of 6 downregulated LE lncRNAs by LASSO algorithm and corresponding expression levels to construct risk model (the risk score formula: Risk score = coefficient1∗value1 + coefficient2∗value2 + coefficient3∗value3+…+ coefficient n∗value n , where value refers to expression level of selected downregulated LE lncRNAs) Therefore, HCC patients from TCGA database were divided into high- and low-risk group on the basis of the median risk score. Univariate and multivariate Cox regression analyses were utilized to identify whether age, gender, grade, stage, and risk score can be regarded as independent prognostic factors for HCC patients. After, we performed the Kaplan-Meier survival curves to compare the survival outcomes of high- and low-risk group. Then, receiver operating characteristic (ROC) curve analysis was performed to test the sensitivity and specificity of the prognostic risk score model for survival in HCC patients. The area under the ROC curve (AUC) was derived as reported previously. And principal-component analysis (PCA) was performed to verifies the grouping ability of risk model.

A prognostic nomogram based on risk score and traditional clinical factors (including age, gender, stage, and TNM classification) was constructed to make a quantitative prognostic prediction of HCC patients. At the same time, the concordance index (C-index) and calibration curves of 1-, 3-, and 5-year were performed to validate the reliability and accuracy of the prognostic nomogram.

By the Limma package from Bioconductor in R, a lncRNA-mRNA co-expression was constructed. Pearson correlation analysis was used to calculate the co-expression relationship between 6 downregulated LE lncRNAs and protein coding gene. The mRNAs correlated to 6 downregulated LE lncRNAs were identified using Pearson correlation analysis. |Pearson correlation coefficient| >0.5 and p<0.05 was considered significant. The Database for Annotation, Visualization and Integrated Discovery (DAVID) Bioinformatics Tool (version 6.8) was used to perform GO and KEGG functional enrichment analysis.

**Table S1. Sequences of primers and siRNA used in the study**

| **Name** | | **Sequence (5’-3’)** |
| --- | --- | --- |
| LINC02428-F | TGGCACATACAGAGGAAAAGAC | |
| LINC02428-R | TTTCTCATAATTCTGGAAGCAAG | |
| IGF2BP1-F | CAAAGGAGCCGGAAAATTCAAAT | |
| IGF2BP1-R | CGTCTCACTCTCGGTGTTCA | |
| KDM5B-F | AGTGGGCTCACATATCAGAGG | |
| KDM5B-R | CAAACACCTTAGGCTGTCTCC | |
| GAPDH-F | ACAACTTTGGTATCGTGGAAGG | |
| GAPDH-R | GCCATCACGCCACAGTTTC | |
| Si-KDM5B-1 | GCCAUCUCCUGUUCUUGUATT | |
| Si-KDM5B-2 | GGAGCUGACAUUGCCUCAATT | |
| ChIP-primer-F | CCCAGTTTTCGCCCTGTTCA | |
| ChIP-primer-R | AGGCCAACGAGTTTCTAGCG | |

**Table S2. Specific probes for FISH assay**

| **Name** | | **Sequence (5’-3’)** |
| --- | --- | --- |
| LINC02428-probe | T+TCCCAATATCCAGAGTCAG+TCTCCTCATATCTCC+TGCTGAGGT+TTTATGACAACCCAA+TATCCAGAGTCAG+TCTCCTCAT | |
| 18S- probe | CTGCCTTCCTTGGATGTGGTAGCCGTTTC | |
| NC-probe | TGCTTTGCACGGTAACGCCTGTTTT | |

**Table S3. 49 downregulated LE lncRNAs**

| LINC01093 | AC010280.2 | AC099508.2 | AC104809.1 | LINC02027 | HAO2-IT1 | AL021328.1 |
| --- | --- | --- | --- | --- | --- | --- |
| LINC02428 | AL161668.4 | FAM99A | AC106822.1 | AP000851.2 | AC020978.4 | FAM99B |
| AC008549.1 | LINC01767 | PCDH9-AS2 | LINC00907 | AC099508.2 | AL138749.1 | AP006285.2 |
| AL161740.1 | LINC01018 | LINC01831 | TMEM220-AS1 | LINC02037 | LINC01702 | AP003716.1 |
| LINC02362 | LINC00261 | LINC01714 | HNF4A-AS1 | U91324.1 | AL391095.2 | LINC01348 |
| AC105105.2 | AC009137.2 | U91319.1 | CPS1-IT1 | AC018467.1 | AC016395.1 | LINC01370 |
| AC005304.2 | AC115619.1 | AP001065.1 | LINC02499 | AL354872.2 | AL592494.1 | AC087273.1 |


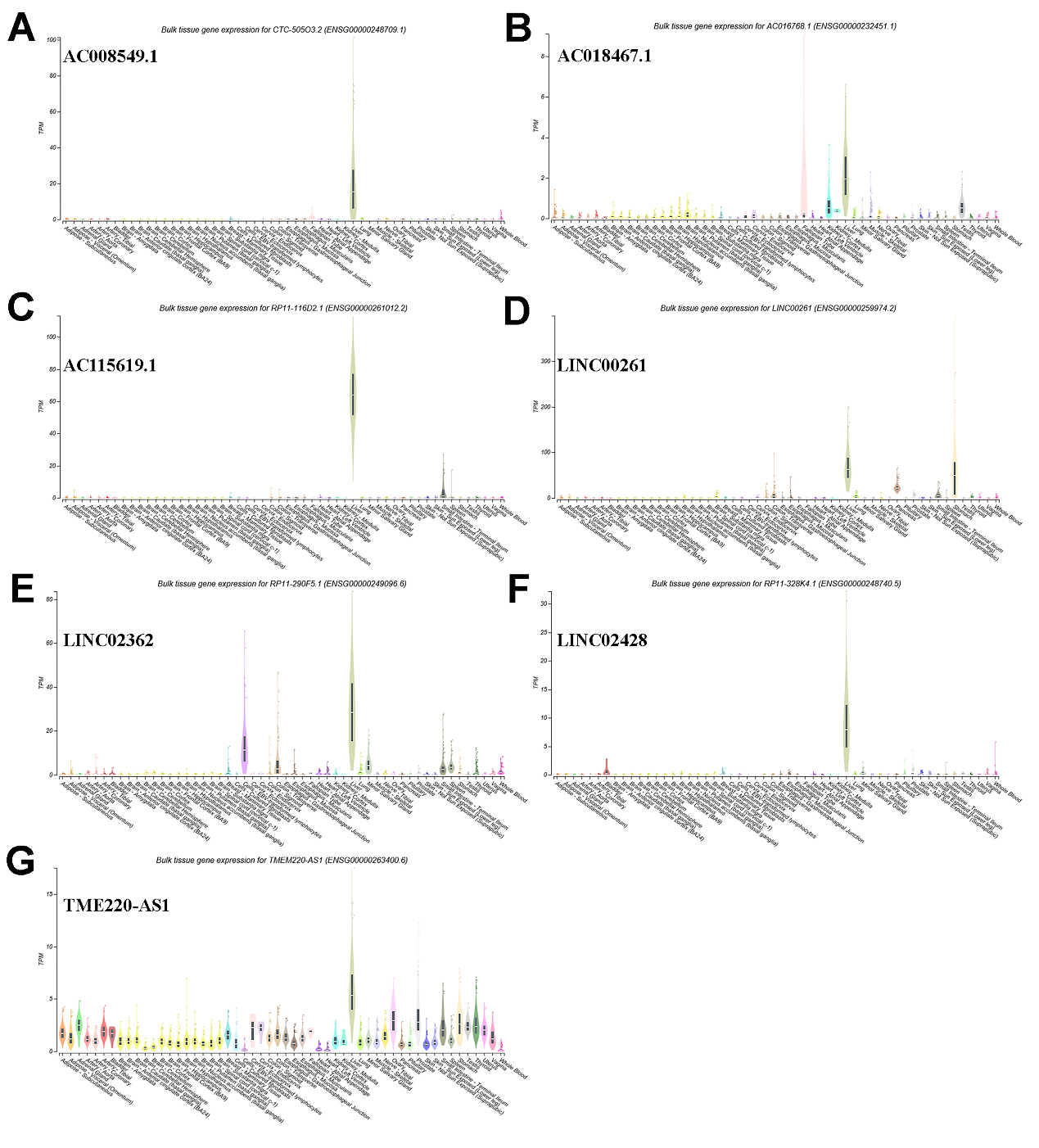


**Figure S1** Downregulated LE lncRNAs expression in 53 normal tissues from the GTEx database. **(A-G)** The expression of the downregulated LE lncRNAs AC008549.1, AC018467.1, AC115619.1, LINC00261, LINC02362, LINC02428 and TMEM220-AS1in 53 normal tissues.


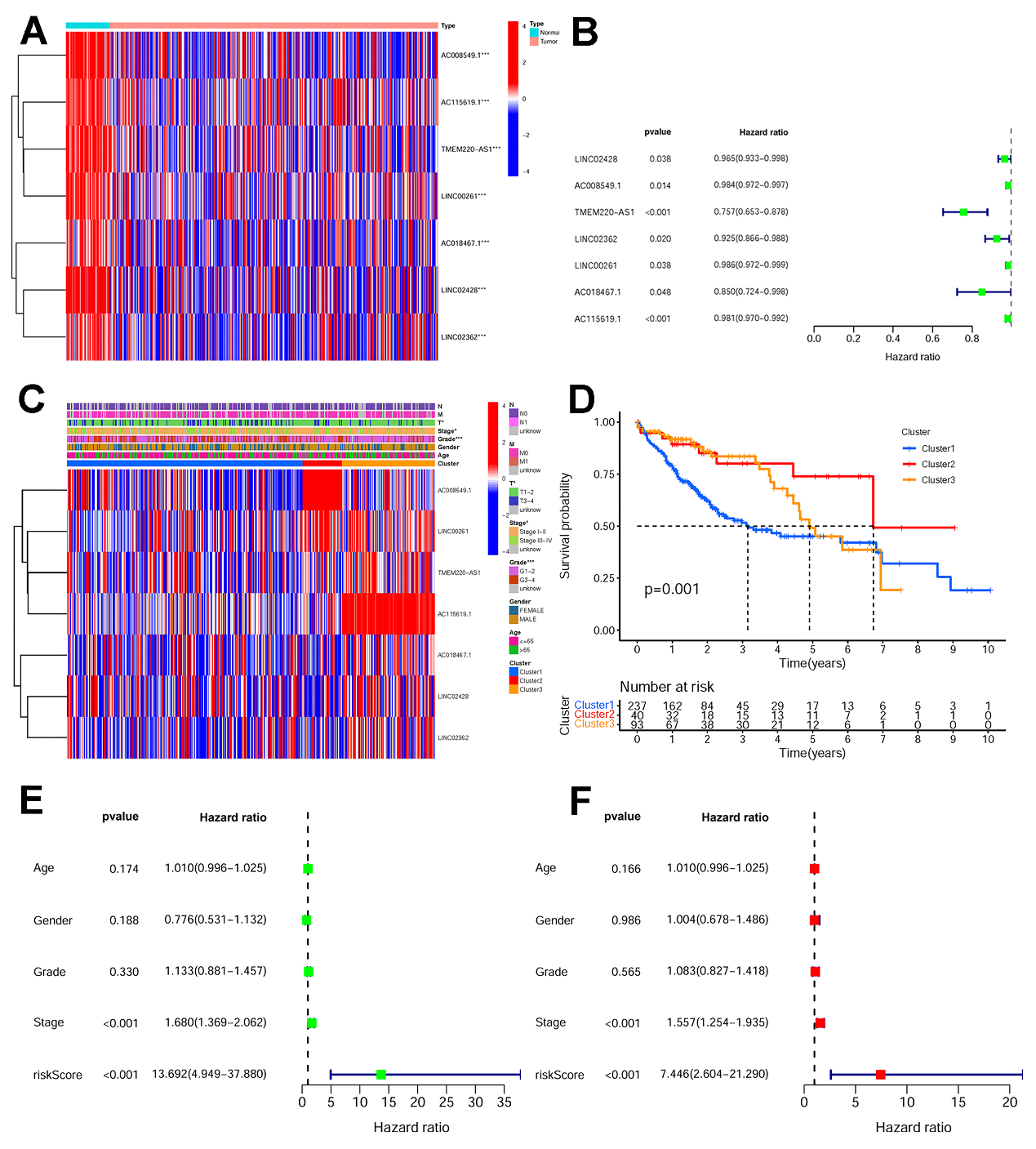


**Figure S2** Consensus clustering analysis of downregulated LE lncRNAs. **A** The heatmap about expression of seven downregulated LE lncRNAs in normal liver tissues and patients whit HCC from the TCGA database. **B** Forest plot of the univariate Cox regression analysis for the seven downregulated LE lncRNAs. **C** The heatmap of downregulated LE lncRNAs expression and clinicopathological characteristics in different clusters. **D** The survival curves of patients with HCC in three clusters. **E** and **F** Risk scores and clinicopathological characteristics as independent prognostic factoers by univariate and multivariate Cox regression analyses.


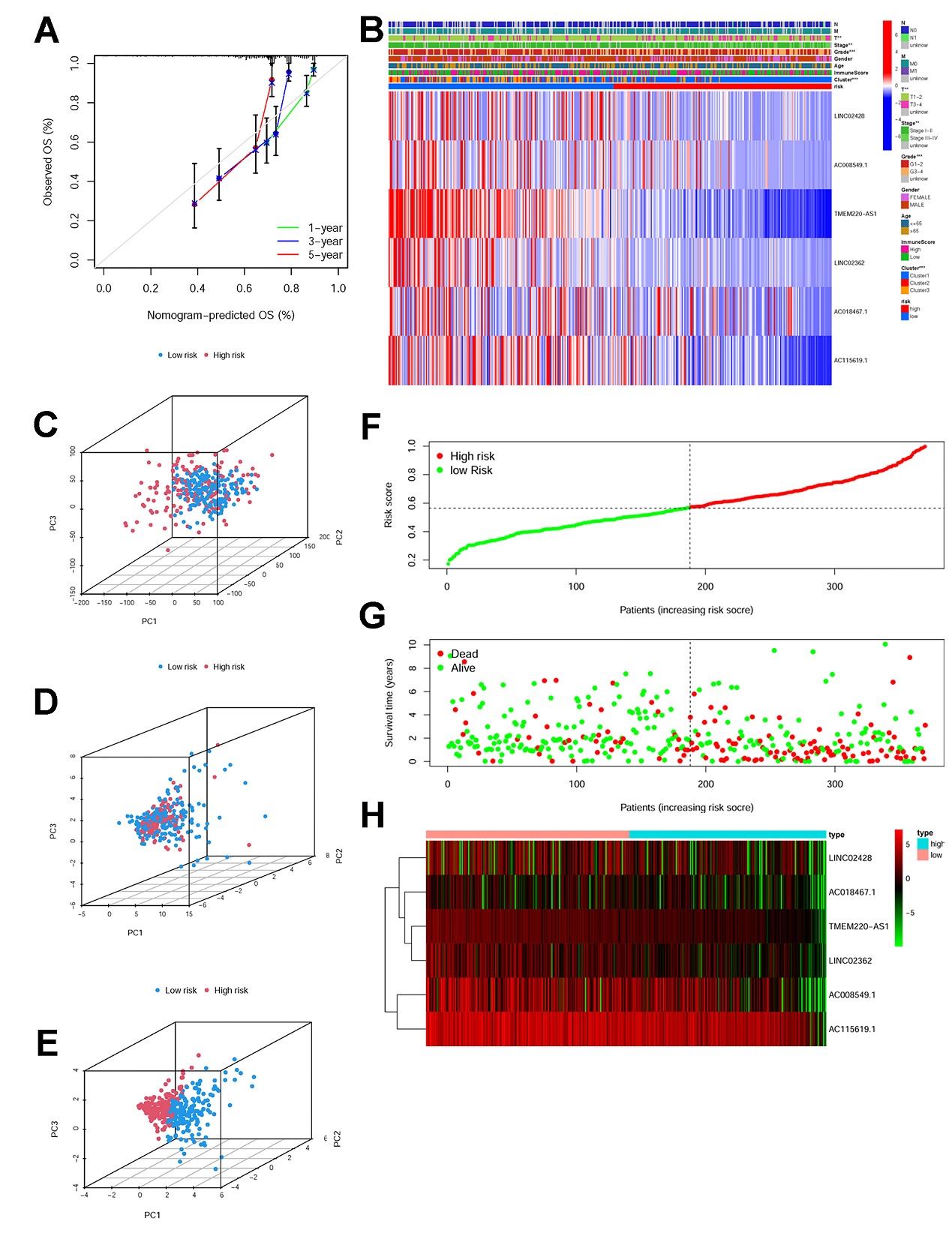


**Figure S3** Establishment and validation of six downregulated LE lncRNAs signatures. **A** The calibration plot of the predictive ability for1-, 3 -, and 5-year survival in patients with HCC. **B** The heatmap of clinicopathological features, immune scores, clusters, risk scores and 6 downregulated LE lncRNAs expression. **C-E** PCA plot of all genes, 49 LE lcnRNAs, and 6 LE lncRNAs. **F-G** Distribution of risk scores, survival status, and 6 LE lncRNAs expression profiles of HCC samples. (*P < 0.05, **P < 0.01, ***P<0.001)


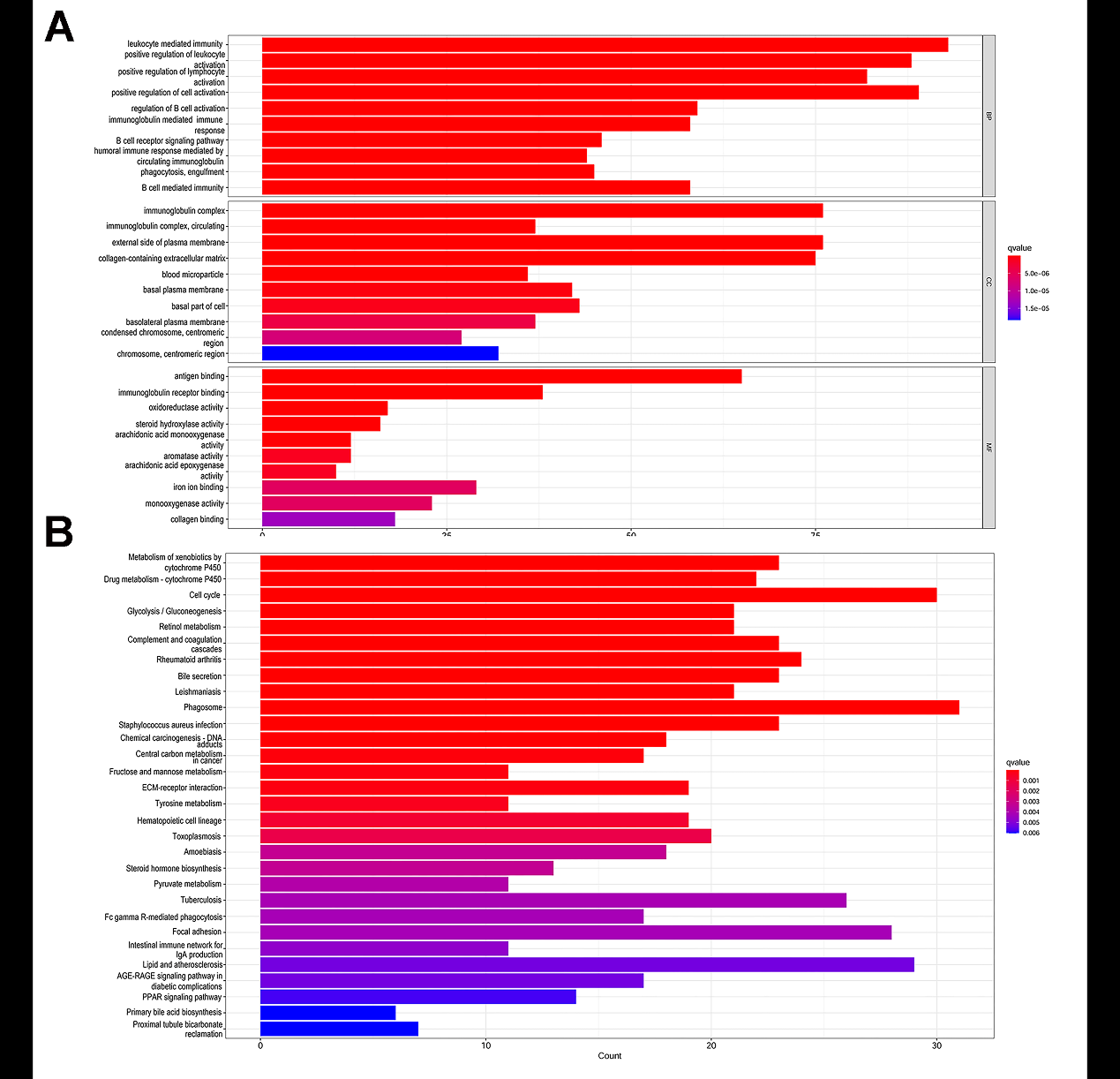


**Figure S4** GO and KEGG functional enrichment analysis of 6 LE lncRNAs. **A** Top 30 significantly enriched GO terms for co-expressed protein-coding genes based on the 6 downregulated LE lncRNAs. **B** The top 30 significantly enriched KEGG pathways for co-expressed protein-coding genes based on 6 downregulated LE lncRNAs.


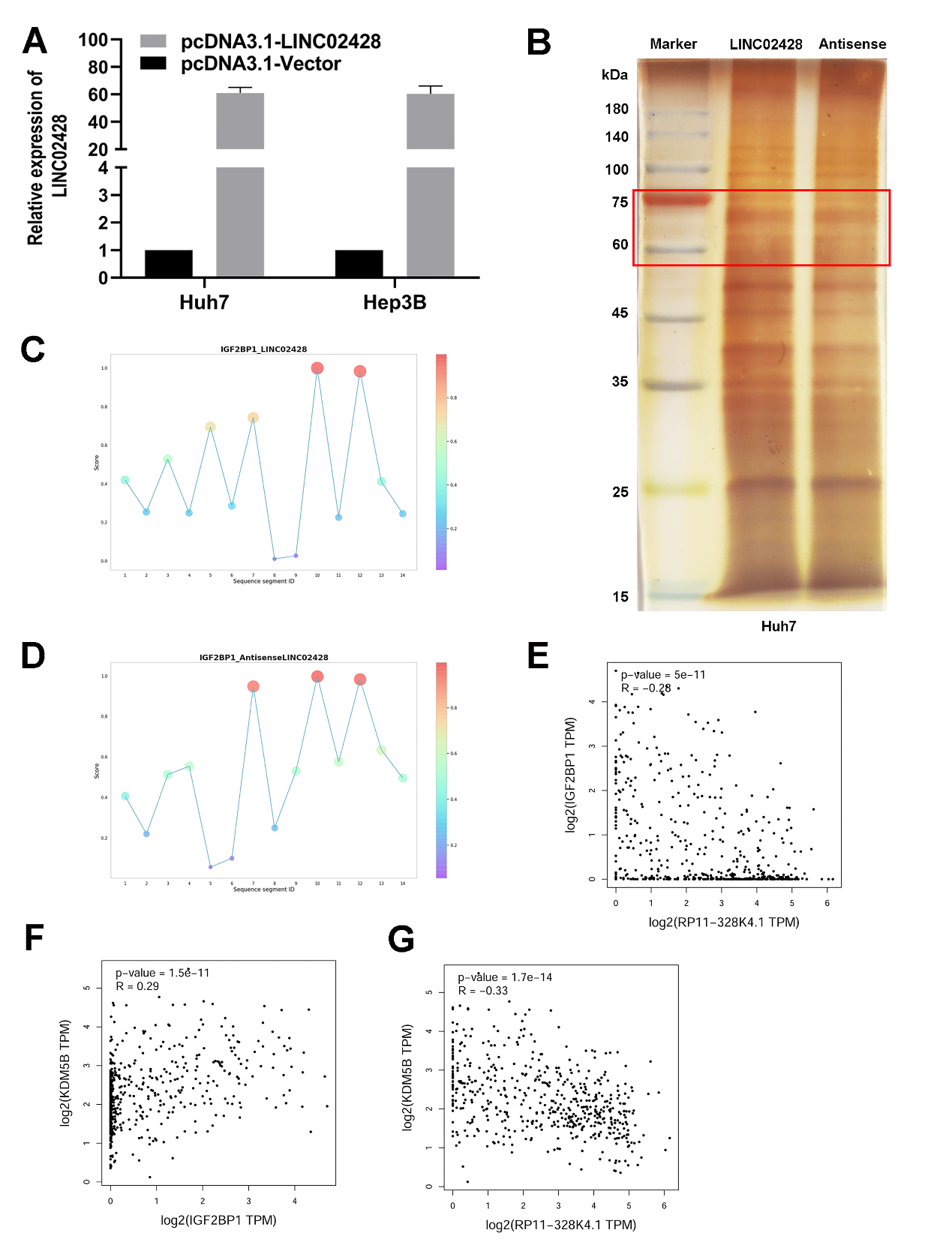


**Figure S5** The KDM5B/IGF2BP1 positive feedback loop in HCC. **A** qRT-PCR showing LINC02428 upregulation in Huh7 and Hep3B transfected with plasmids overexpressed LINC02428. **B** Silver staining of RNA pulldown assay elutes for LINC02428 sense and antisense strands in Huh7 cells. Prediction using the RBPsuite website showed that both LINC02428 sense **(C)** and antisense strands **(D)** could bind to IGF2BP1. The GEPIA database indicated that IGF2BP1 was negatively correlated with LINC02428 **(E)** and was positively correlated with KDM5B **(F).** LINC02428 was negatively correlated with KDM5B **(G)**. (Alias of LINC02428 is RP11-328K4.1)


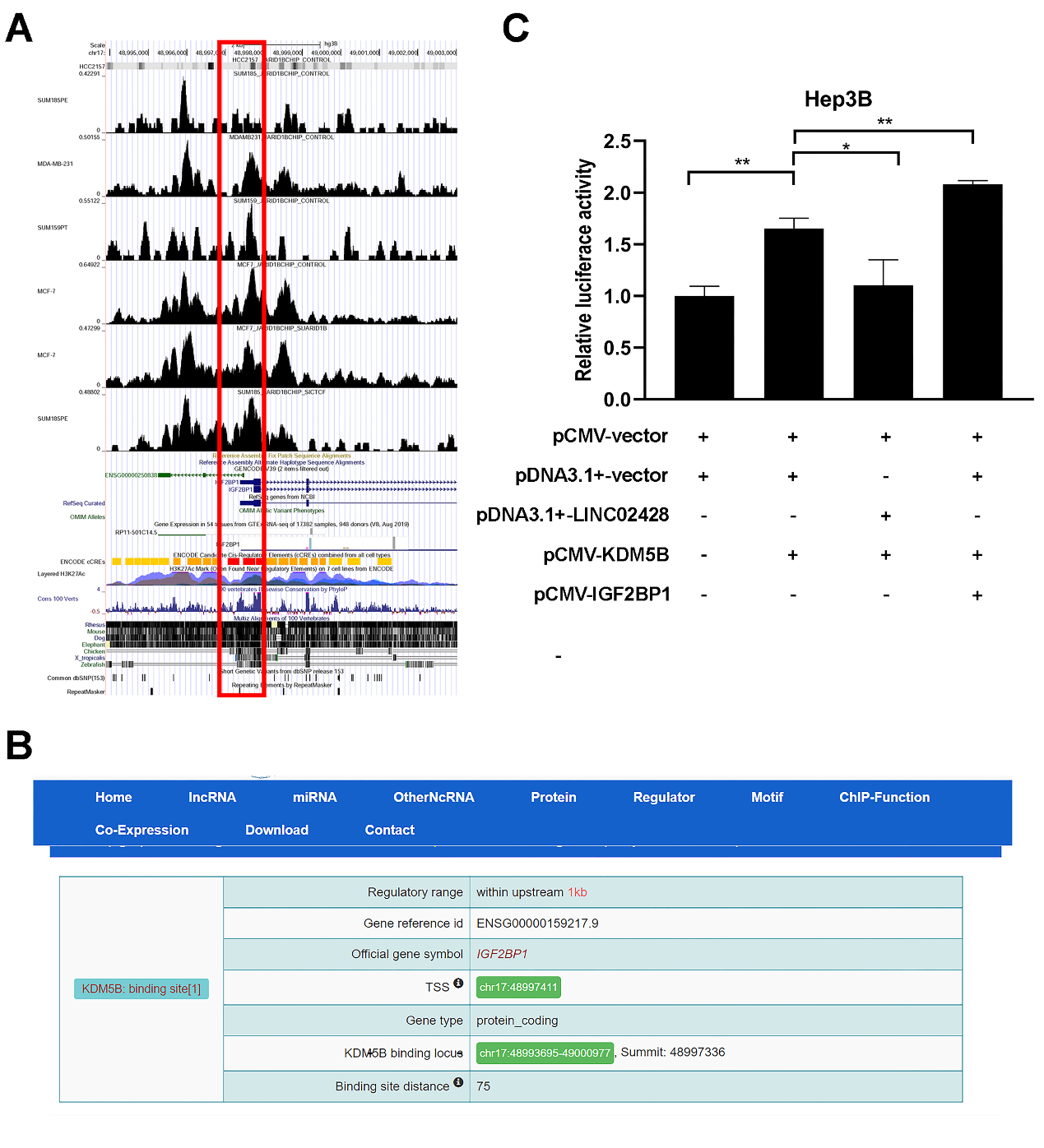


**Figure S6** KDM5B bound to the promoter of IGF2BP1 and regulated the transcription of IGF2BP1. **A** The UCSC database showed the peak enrichment area of KDM5B in the promoter region of IGF2BP1 from ChIP-seq of the Cistrome Data Browser database, and the red box is the peak area we selected for designing ChIP primers . **B** Prediction of KDM5B binding to the IGF2BP1 promoter region using ChIPBase v2.0. **C** Dual-luciferase reporter assay proved that KDM5B bound to the IGF2BP1 promoter to promoted its transcription, and IGF2BP1 transcription promoted KDM5B could be enhanced by IGF2BP1 upregulation or decrease by LINC02428 overexpression in Hep3B cells.


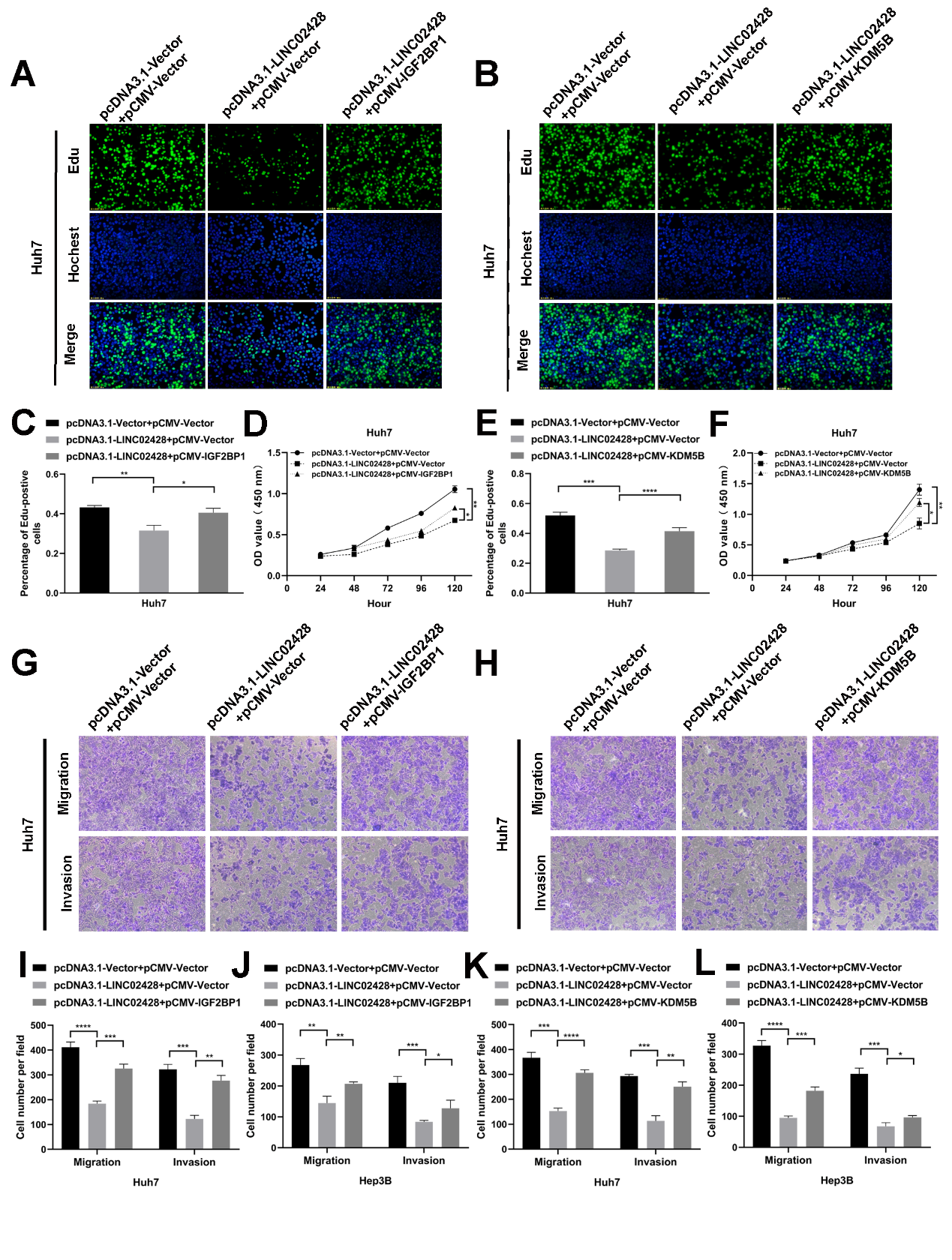


**Figure S7** IGF2BP1 or KDM5B rescued anti-tumor phenotypes induced by overexpression of LINC02428. **A, C** and **D** EdU and CCK-8 assays of rescue experiement about IGF2BP1 and LINC02428 in Huh7 cells. **B, E** and **F** rescue experiement between KDM5B and LINC02428 by EdU and CCK-8 assays in Huh7 cells. Migration and invasion rescue assays indicated IGF2BP1**(G, I** and **J)** or KDM5B **(H,** **K** and **L)** overexpression rescued inhibition of migration and invasion generated by LINC02428 upregulation.
